# Immunometabolism: Towards a Better Understanding the Mechanism of Parasitic Infection and Immunity

**DOI:** 10.3389/fimmu.2021.661241

**Published:** 2021-05-27

**Authors:** Jing-yue Chen, Ji-kai Zhou, Wei Pan

**Affiliations:** ^1^ Jiangsu Key Laboratory of Immunity and Metabolism, Department of Pathogen Biology and Immunology, Xuzhou Medical University, Xuzhou, China; ^2^ The First Clinical Medicine, Xuzhou Medical University, Xuzhou, China

**Keywords:** parasites, infection and immunity, metabolic reprogramming, immunometabolism, immune regulation

## Abstract

As a relatively successful pathogen, several parasites can establish long-term infection in host. This “harmonious symbiosis” status relies on the “precise” manipulation of host immunity and metabolism, however, the underlying mechanism is still largely elusive. Immunometabolism is an emerging crossed subject in recent years. It mainly discusses the regulatory mechanism of metabolic changes on reprogramming the key transcriptional and post-transcriptional events related to immune cell activation and effect, which provides a novel insight for understanding how parasites regulate the infection and immunity in hosts. The present study reviewed the current research progress on metabolic reprogramming mechanism exploited by parasites to modulate the function in various immune cells, highlighting the future exploitation of key metabolites or metabolic events to clarify the underlying mechanism of anti-parasite immunity and design novel intervention strategies against parasitic infection.

## Introduction

As one of the relatively successful pathogens, parasites infect about one quarter of the world’s population, seriously endangering human health. Many parasites can live in their host for a long time without causing obvious clinical symptoms. The harmonious symbiosis depends on the advanced strategy exploited by parasites to regulate the metabolism and immunity in hosts. On the one hand, the parasites rely on metabolic enzymes to decompose the host’s metabolites or nutrients to meet their own growth, development and reproduction needs (1). On the other hand, they secrete a variety of parasite-derived molecules such as excretory/secretory products to reduce or inhibit host immune response (2). By this way, they resist the host’s anti-infective immunity. Metabolism and immunology were originally considered to be two separate disciplines. However, with the development of holistic integrative medicine, scientists have come to realize that the metabolic changes of immune cells can control the phenotype of immune cells by regulating key transcription and post-transcriptional events related to cell activation, thereby affecting their functions. Immunometabolism, the new frontier discipline, was born, which provides a novel insight for clarifying the pathogenesis of major human diseases including parasitic diseases and propose an emerging target for vaccine and drug development (3).

As a hot research field in recent years, immunometabolism reveals that the proliferation, differentiation and function of immune cells can be directly or indirectly modulated by reprogramming the intrinsic metabolic pathways in immune cells (4). For example, regulating the level of intracellular succinic acid can alter the production of IL-1β in macrophages (5). In addition, different immune cells or even differential stages can have distinguished metabolic phenotypes (6). For example, activation of macrophage through pattern recognition receptors (PRRs), such as Toll−like receptor 4 (TLR4), induces HIF-1α expression to promote glycolysis and pentose phosphate pathways mainly occurring in M1 macrophages, while stimulation of IL-4 induces tricarboxylic acid cycle (TCA cycle, or Krebs cycle) and oxidative phosphorylation (OXPHOS) in M2 macrophages (Reviewed in Reference 7). These findings provide a metabolic insight to explore the underlying mechanism of immune response in order to modulate host immunity to parasites. At present, the research achievements in this field mainly focuses on tumors and metabolic diseases (8–10). However, immunometabolism, as an emerging field, is in its initial stage in parasitic infection and immunity, although researchers have made a lot of studies on the metabolism and immunity in response to parasitic infection, respectively. The present study reviewed the latest advances on how parasites exploit metabolic changes to reprogram the host immunity, in order to provide a novel perspective to further explore the mechanism of infection, immunity and host-parasite interplay in the view of immunometabolism.

**Figure 1:**
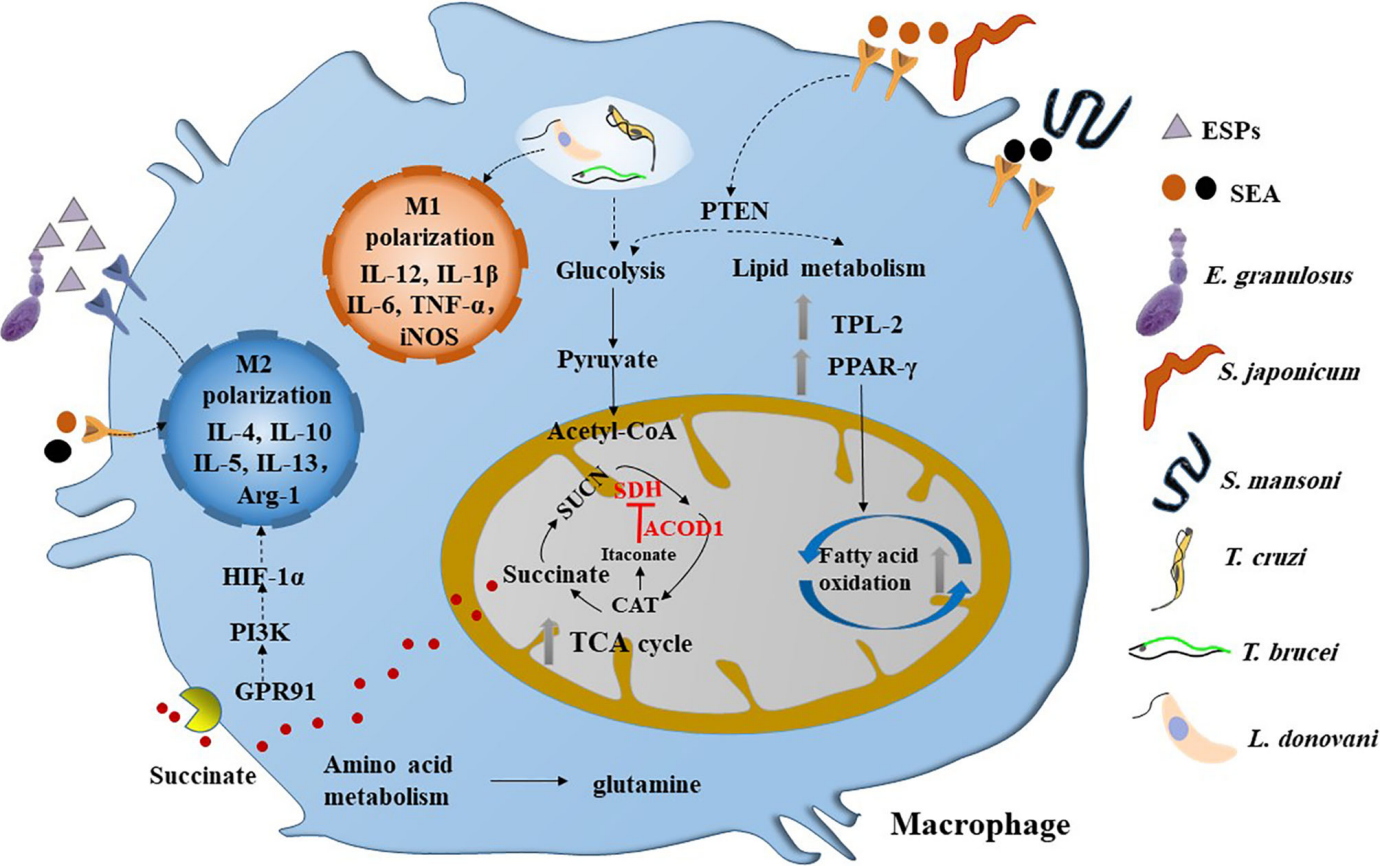
Parasitic infections induce the metabolic reprogramming in macrophages****. M1 type macrophages can be activated by the infection of intracellular parasites such as *T. cruzi*, *T brucei* and *L. donovani*, which are characterized by glycolysis. However, helminths infection can induce the M2 polarization of macrophage in the chronic stage, which are dominated by fatty acid oxidation and broken of TCA cycle. The SEA of *Schistosoma* sp. can inactivate PTEN and its downstream pathway, thereby promoting glycolysis and fatty acid oxidation and inducing a mixed but type 2 biased anti-inflammatory response in macrophage. Interestingly, the ESPs of larval *E. granulosus* can activate the expression of ACOD1, which can encodes the enzyme that catalyzes the cis-aconitate to produce itaconate in the TCA cycle. It is reported that itaconate can inhibit the activity of SDH, thereby leading to the accumulation of succinate. Moreover, the succinate can activate GPR91 to induce the M2 polarization of macrophage through PI3K/HIF-1α pathway. ESPs, excretory/secretory products; SDH, succinodehydrogenase; GPR91, G-protein coupled receptor 91; SEA, soluble egg antigens; CAT, cis-aconitate; HIF-1α, hypoxia-inducible factor 1α; PTEN, phosphatase and tensin homolog; TPL-2, tumor progression locus 2; PPAR-γ, peroxisome proliferator activated receptor-γ.

Helminths and protozoa belong to extracellular and intracellular parasites, respectively. They induce distinguished immune responses in host cells. For example, in genetically resistant murine models of *Leishmania*, the infection polarizes macrophages in lesions towards a classically activated M1 state. These macrophages secrete inflammatory mediators (*e.g.* IL-12, TNF-α) and express high levels of iNOS and NADPH oxidase (NOX2), leading to production of nitrous oxide (NO) and reactive oxygen species (ROS), respectively, with concomitant containment or clearance of the parasites (**Figure 1**). Moreover, the progression of self-resolving lesions is linked with a strong CD4^+^T cell helper (Th1) immune response and production of proinflammatory cytokines, IFN-γ and IL-12 (11). In contrast, non-resolving infections are accompanied with a Th2 immune response and production of anti-inflammatory cytokines (*e.g.* IL-4, IL-13 and/or IL-10), and polarization of activated M2 states that promote tissue repair and constitute a permissive host cell reservoir (12).

However, the helminths are considered to be the strongest natural inducers of type 2 immunity. It is well-known that in the early infection stage, the host immune system is rapidly mobilized to induce immune characteristics dominated by T-helper 1 (Th1) immune response, producing a large number of inflammatory factors (IFN-γ, IL-6, TNF-α, IL-12, IL-17A, *etc.*) to help kill or eliminate parts of helminths. However, with the release of a large number of parasite-derived molecules, T-helper 2 (Th2) immune response and a large number of anti-inflammatory factors (IL-4, IL-10, TGF-β, *etc.*) is triggered, which can inhibit or down-regulate the anti-infective immunity of the host, contributing to the long-term survival of worms in host (13). There are numerous studies have shown that helminth derived excretory-secretory products (ESPs) act as the key modulators to regulate the immune responses mentioned above (2). Moreover, recent studies also show that the helminth ESPs can induce selective activation of macrophage and DC cells, inhibit the differentiation of Th1 and Th17, and promote the production of immunosuppressive cell subsets such as regulatory T cells (Tregs) and regulatory B cells (Bregs) (14–16), thus maintaining a long-term or chronic infection state. For supporting these findings, mounting epidemiological evidence shows that helminth infections inversely correlate with many autoimmune, inflammatory, and importantly, metabolic diseases (17–20). Furthermore, animal studies have also shown that parasitic infection and their derived molecules can prevent the progression of these diseases (21–25).

Interestingly, it is increasingly recognized that host nutritional status can have a major influence on anti-parasite infection and immunity in part because nutrient and pro-inflammatory signals are integrated through common evolutionarily conserved signal transduction molecules (3, 26). Taken together, these studies highlight the intimate connectivity between parasite, immunity and metabolism in hosts, the need to investigate these interactions *in vivo* and the potential to exploit the specific metabolic signaling pathways in developing new intervention strategies against human disease including parasitic diseases. In the following text, we mainly focused on how parasites trigger the metabolic reprogramming to control the differential and effector function in macrophage, dendritic cells, T and B cells in hosts.

## Macrophage

### Helminth Parasite Infections Induce M2 Macrophage Polarization and Lipid and Glucose Metabolic Reprogramming in Macrophage

Macrophage is an important component of the innate immune system, which acts as the first line of defense against parasitic infection. It is well-known that macrophage work as antigen-presenting cells to uptake, process and present parasitic antigens, initiating and inducing adaptive immune response. Macrophage can be divided into M1 and M2 types, exhibiting distinguished metabolic characteristics (27). M1 macrophage is shown to increase the expression of glycolysis and pentose phosphate pathways, while M2 macrophage is induced after the stimulation of IL-4 and maintains mitochondrial respiration and OXPHOS (Reviewed by Reference 7). In the chronic or late stage of helminth infection, the host immune response mainly belongs to type 2 immune response, in which M2 macrophage is dominant (**Figure 1**). Helminth infection promotes IL-4 release and maintains the OXPHOS of macrophage so as to differentiate into M2 macrophages (28–30). It can be seen that the changes of metabolism in immune cells are not only determined by oxygen and nutrition, but also driven by immune signaling molecules. Huang et al. found that during the infection of *Helicopteroides polygyrus* (*H. polygyrus*), fatty acid oxidation (FAO) provides raw material for the TCA cycle in M2 macrophages. Moreover, inhibition of lipolysis suppressed M2 macrophage activation during parasite infection and blocked protective responses to this pathogen (31). M2 macrophages can better support cell survival, so that macrophage can continue to resist parasites for a relatively long time. In addition, it has been reported that following *S. mansoni* infection, tumor progression locus 2 (TPL-2), a MAP3 kinase, can regulate the oxidative lipid metabolism which is essential for M2 macrophage activation. It can regulate type 2 inflammation, immunopathology and hepatic fibrosis by supporting lipolysis and activation of M2 macrophages, thus providing a way for treating inflammatory diseases (32). It is noteworthy that Leonie et al. found that chronic helminth infection promotes adipose tissue M2 macrophages without reducing M1 gene expression or cell numbers (33). Similarily, Cortes-Selva et al. found that *S. mansoni* infection induces M2 polarization in macrophage to improve hyperlipidemia and atherosclerosis (34). These data suggest that helminth infection induce M2 polarization and metabolic reprogramming in macrophage.

Furthermore, phosphatase and tensin homolog (PTEN), a well-known tumor suppressor gene, functions as a modulator of glucose and lipid metabolism in tumor cells (35), has been recognized to involve in parasitic infection and related pathogenesis (**Figure 1**). Our previous study recently showed that the soluble egg antigen (SEA) of *Schistosoma japonicum* (*S. japonicum*) induces metabolic reprogramming in macrophage, which is mainly manifested by the upregulated expression of genes related with glycolysis pathways and fatty acid oxidation (36). Moreover, this phenotype is also observed in the fibrosis liver and colon following *S. japonicum* infection (36, 37). In addition, we demonstrated that over-expressed PTEN in the colonic cell line CT-26 can inhibit the activation of glucolipid metabolism induced by SEA (37) (**Figure 1**). These studies not only suggest that glucose and lipid metabolic remodeling underpins the capacity of macrophage function, but also indicate that targeting PTEN may prevent the fibrosis induced by *Schistosoma* sp. Besides, other underlying metabolic events related with the infection of the parasite have been reviewed by Diana and Keke [As reviewed in Reference (38)].

### Intracellular Parasites Inhibit M1 Macrophage Activation to Resist Anti-Infective Immunity *via* Complex Metabolic Events

Activation of M1 macrophage is essential to clear or inhibit the intracellular parasites in hosts. Recent studies in *Leishmania* have shown that metabolic reprograming of M1 macrophage underpins the capacity of these cells to act as permissive or non-permissive host reservoirs [As reviewed in Reference (27)]. It is well established that resting macrophage can differentiate into M1 macrophage after being activated by lipopolysaccharide (LPS). The M1 activated cells show increased glycolysis rate, activated pentose phosphate pathway, and decreased OXPHOS level, which is known as Warburg effect or aerobic glycolysis. Because OXPHOS is inhibited, the higher levels of the TCA intermediates were accumulated in the cells, which in turn mediate the immune response in macrophage (**Figure 1**). For example, succinate in macrophage can promote the production of IL-1β by up-regulating HIF-1α (5). Pyruvate Kinase M1 (PKM1) is a rate-limiting enzyme in the conversion of phosphoenolpyruvate to pyruvate in glycolysis. PKM2, PKM1’s isoenzyme, exists in the form of monomer or dimer and can translocate to the nucleus and interact with HIF-1α (39). Activation of PKM2 can induce a series of changes in macrophage cells. The changes contain down-regulation of HIF-1α, IL-1β, glycolysis, pentose phosphate pathways and succinate level. It attenuates an LPS-induced pro-inflammatory M1 macrophage phenotype while promoting typical traits of an M2 macrophage. The increase of M2 cytokine IL-10 further supports this hypothesis (40). 6-phosphogluconate dehydrogenase (PGD) is a key enzyme in pentose phosphate pathway. Koo et al. found that macrophage infected with *Trypanosoma cruzi* (*T. cruzi*) have lower levels of glycolysis compared to classical activated macrophages. Adding 6-aminonicotinamide, the pharmacological inhibitor of PGD, to the co-culture system including macrophage infected with *T. cruzi* and IFN-γ, abolished the nitric oxide (NO) and reactive oxygen species (ROS) production in macrophage, indicating that pentose phosphate pathway participates in early control of parasite replication in macrophage (41). The mammalian target of rapamycin (mTOR), a serine/threonine kinase, can regulate protein synthesis through the phosphorylation of some factors in the proteins translation. mTORC2 is associated with enhanced glucolysis in macrophage, the deletion of mTORC2 hinders the activation of macrophage, with the result of decreasing immunity to a parasitic *nematode* (42). These data suggest that enhancement of glucolysis and pentose phosphate pathways may resist the invading of parasites in early stage of infection.

Intracellular parasites can consume or increase enzymatic substrates to alter the immune function of macrophage. *In vitro* studies have shown that in the presence of L-arginine, macrophage can produce NO through type II NO synthase (NOS-II) to kill *Trypanosoma brucei* (*T. brucei*) (43, 44). In the course of *T. brucei* infection, the immune effect of macrophage on worms was down-regulated by consuming L-arginine and inhibiting NO production, because the arginase (ARG) activity was up-regulated. ARG competes with NOS-II for their common substrate (45). Study has showed that the ARG activity in susceptible mice was higher than that of anti-Trypanosoma mice, and NO production and trypanosome killing were increased when ARG was specifically inhibited (46). Cruzipain, a parasite antigen, induces ARG-1 expression which is beneficial to the replication of parasites, while inhibition of cruzipain restricts the growth of parasites (47). In addition, cruzipain inhibits the activation of macrophage during early infection, and no significant IL-12 expression occurred in infected macrophage (48). As a positive stimulator of inflammation, LPS can up-regulate M1 by activating glycolysis pathway (5). Interestingly, *T. brucei* metabolite indolepyruvate can inhibit this effect and reduce HIF-1α levels, resulting in a decrease in production of the pro-inflammatory cytokine IL-1β (49). Thus, indolepyruvate down-regulates innate immunity to mediate immune escape by inhibiting glycolysis and HIF-1α (49). Ecto-nucleoside triphosphate diphosphohydrolase (E-NTPDase) belongs to the ecto-nucleotidases family. The enzyme hydrolyzes nucleotides triphosphate and/or diphosphate into monophosphate products, which are subsequently hydrolyzed into adenosine. It is important for parasite nutrition by facilitating acquisition of extracellular purines. Peres et al. found that E-NTPDase was expressed in *Leishmania infantum* (*L.infantum*), including on the cell membrane. In addition, inhibiting E-NTPDase could decrease the number of *L. infantum* in macrophage (50).

Studies in recent years have uncovered that the TCA cycle can reprogram the metabolic flux, leading to the accumulation of metabolites such as succinate and fumarate, which in turn act as signaling molecules to guide macrophage function (51). For example, succinate has been demonstrated to modulate the immune response in macrophage *via* interacting with its receptor G-protein coupled receptor 91 (GPR91; also known as succinate receptor 1, SUCNR1) (52, 53). Interestingly, the emerging evidence in obesity and tumors show that SUCNR1 acts as the vital anti-inflammation node in macrophage (54, 55). It is reported that *T. cruzi* can produce elevated level of succinate before invasion of host cells (56). However, it is still known whether the produced succinate is associated with the immune remodeling in parasitic infection. Another example is itaconate, which is one of the most easily induced metabolites when macrophage activation (57). Lampropoulou et al. found that itaconate exerts anti-inflammatory effects by inhibiting succinate dehydrogenase-mediated oxidation of succinate (58). It is worth to investigate if parasites can regulate itaconate/succinate axis to reprogram the polarization of macrophage. In addition, glutamine (Gln) is highly utilized in mice macrophage (59), which is also essential in the proliferation and immune effect of macrophage. Parry-Billings et al. reported that the decline concentration of Gln greatly decreased the proliferation rate of human lymphocytes and the phagocytosis of mouse macrophage (60). Palmieri et al. found that pharmacologic inhibition of Gln synthase decreases Gln in cell and skews M2-polarized macrophages toward the M1-like phenotype (61). Moreover, Jha et al. confirmed that Gln deprivation inhibited M2 polarization and CCL22 production (62). Gln promotes M2 polarization through a glutaminolysis-derived a-ketoglutarate-dependent pathway that also inhibits M1 polarization by suppressing the NF-κB pathway (63). Therefore, macrophage polarization and function are triggered by complex metabolic events, which may be utilized by parasites to down-regulate the anti-parasite immunity.

### M2 Polarization of Macrophage Is Likely the Underlying Mechanism Exploited by Helminths to Alleviate Metabolic Diseases

The long-term survival in hosts underpin the capability of helminths to manipulate host immune and metabolism, which is increasingly recognized to be an effective strategy against metabolic disorder associated diseases. The “metabolic” activation of macrophage produces low-grade persistent inflammation, which intersects in the development of obesity and its related syndromes (64–66). In obese mice, M1 macrophages are accumulated, while parasitic infection often induce M2 polarization of macrophage, thereby reducing the inflammatory response (2). Lu et al. showed that infection of the larval *Echinococcus granulosus* (*E. granulosus*) can enhance the lipolysis in the fat of mice, which accompanies with enhanced arginine metabolism and PPAR-γ pathway activation (67). It is well known that arginine metabolism is closely associated with M2 polarization. There is no doubt that a beneficial effect of parasitic infection or its derived molecules is observed in metabolic diseases (23–25). In an obese mice model, we recently found that the ESPs supplementation of the larval *E.granulosus* can effectively suppress the activation of the macrophages in colon as well as the neuroinflammation induced by microglia and astrocytes in brain, and notably improve the obesity-induced cognitive impairment. Moreover, van der Zande and colleagues found that one of the major *S mansoni* immunomodulatory glycoproteins, omega-1, improves metabolic homeostasis in obese mice in independent inhibition of food intake (68). Therefore, utilizing the M2 polarization effect of helminths provides a new insight to prevent metabolic diseases.

## Dendritic Cells

As the most powerful antigen presenting cells, dendritic cells (DC) recognizes the molecular patterns (PAMPs) associated with these parasites through TLR, and presents the parasite derived antigens to T cells, thereby mediating the cellular immune response (69, 70). DC can be divided into resting stage, differentiation stage and activation stage. Under the stimulation of granulocyte-macrophage colony-stimulating factor (GM-CSF) and IL-4, monocytes derived from peripheral blood can differentiate into DCs accompanied by increased expression of peroxisome proliferator activated receptor-γ (PPAR-γ) and PPAR-γ co-activator 1α (PGC1α). PPAR-γ is the key transcription factor to regulate lipid metabolism, and PGC1α is the main regulator of mitochondrial biosynthesis (71, 72). Increased expression of them leads to increased mitochondrial biosynthesis (73). Rotenone, an inhibitor of mitochondrial respiration in monocytes, inhibits DC differentiation (74, 75). In these studies, increased citrate synthase activity was closely related to DC differentiation. Citrate produces isocitric acid and α-ketoglutarate in TCA cycle. Citrate is also exported from mitochondria and converted into acetyl-CoA, which is the precursor of fatty acid synthesis, thereby regulating fatty acids synthesis. The differentiation of monocytes to DC *in vitro* and the development of DC in lymphoid organs and peripheral tissues both depend on fatty acid synthesis (76). Thus, it can be concluded that DC differentiation depends on the coordination of mitochondrial function and fatty acid synthesis. In line with these findings, we showed that both *S. japonicum* and its egg soluble antigens can upregulate the mRNA expression of the enzymes related with both fatty acid synthesis and oxidation in liver and colon (36, 37). It is still unclear if these changes could remodel the function of DC.

Furthermore, Falcon reported that the ESPs from *Fasciola hepatica* (F. hepatica) induces tolerogenic properties in DC. It is characterized by increased IL-4, IL-5, IL-10 and TGF-β production (77). DC with tolerogenic properties exhibits distinct metabolic characteristics. Malinarich et al. found that the expression of OXPHOS-related genes is upregulated in tolerogenic DC. The expression of genes related to FAO is also increased in the process. It is reported that inhibition of FAO can prevent the function of tolerogenic DCs (78). AMP-activated protein kinase (AMPK) is regulated by AMP and has the function of regulating metabolism. Activation of AMPK is shown to weaken anabolic activity, while enhancing the ATP production and body decomposition. Baghdadi showed that the activation of AMPK reduces DC’s ability to produce antigen-peptide-MHC complex, maintaining a tolerogenic phenotype (79). Moreover, Tan showed that adiponectin can stimulated the anti-inflammatory IL-10 production by activating the AMPK dependent pathway to inhibit the activation of DC (80). Thus, AMPK, as a regulator of metabolism, can promote FAO and other catabolism, contributing to the tolerogenic DC. In addition, Tan found that microfilariae of *Brugia malayi* (*B. malayi*) inactivates the mTOR pathway to inhibit DC function (81). On one hand, it downregulates the phosphorylation of mTOR and its regulatory proteins, which leads to the blockage of DC protein synthesis. On the other hand, it upregulates phosphorylated Beclin 1 which is known to play an important role in both autophagosome formation and autolysosome fusion to upregulate autophagy (81). Similarily, Semnani found that the ESPs of *B. malayi* induce DC autophage, inhibit their ability to secrete IL-12 and IL-10 and activate CD4 T^+^ Cells. These data suggest that *B. malayi* induces an orchestrated response in DC that leads to a diminished capacity to function appropriately, which in turn has significant consequences for CD4^+^ T cells (82). These studies supported that parasitic infection induces the reprogramming in DC.

Stimulated by TLR agonists, activated DC showed increased glycolysis, decreased OXPHOS and FAO (83, 84). 2-deoxyglucose (2-DG) is an inhibitor of the key enzyme hexokinase (HK) in glycolysis. Adding 2-DG can strongly block the DC activation process (83, 85). Thus, an increase in aerobic glycolysis is required for the activation and function of DCs. Everts et al. found that OXPHOS is inhibited by NO and that the switch to glycolysis is a survival response that serves to maintain ATP levels when OXPHOS is inhibited (86). GPR91 functions as a receptor for succinate, and GPR91 expression is found mainly in kidney, liver, spleen and small intestine. It is reported that DCs can sense the level changes of extracellular succinic acid through GPR91. Succinate can trigger intracellular calcium mobilization and is able to active DCs in combination with TLR3 or TLR7 (87). The expansion of endoplasmic reticulum and Golgi networks is required for DC activation thus it is necessary to up-regulate the *de novo* synthesis of fatty acids (85). In activated DC, glycolysis is up-regulated and citrate level is increased, which was similar to the differentiation process. Citrate is exported from mitochondria to cytoplasm and decomposed into acetyl-CoA and oxaloacetate. Oxaloacetate further provides a source of NADPH to product ROS and NO, killing pathogens. Acyl-CoA participates in lipid synthesis as raw material (88). Thus, citric acid is the bridge between carbohydrate and fatty acid metabolism, and plays an important role in DC differentiation and activation. Lipids can also affect DC function. Liver is the center of fat metabolism, and immune cells in liver including DC are immune tolerant (89). Ibrahim et al. found that concentrations of lipid in DC is positively correlated with immunogenic versus tolerogenic responses in human and mouse liver (90). But in the case of tumors, high lipids in DC lead to cell dysfunction (91). However, the specific metabolic reprogramming events and their potential role in mediating DC function in response to parasitic infection remain unclear.

## T Cells

In the Th1/Th2 model first described by Mossman and Kauffman, several parasites are considered to be Th2-inducible antigens, which can promote the production of IL-4 and induce the differentiation of CD4^+^ T cells into Th2 (92). Unlike Th1, Th2 cell and its cytokines including IL-4, IL-5, IL-9, IL-10 and IL-13, have protective immune effects against parasitic infection (93). Pesce et al. showed that macrophage-specific Arginase-1 functions as an inhibitor of Th2 cytokine-driven inflammation and fibrosis in infection with the Th2-inducing pathogen *S. mansoni* (94).

Similar to macrophage, activated T cells also exhibit high glucose consumption (95, 96). During T cell activation, it expresses high levels of the glucose transporter 1 (Glut1) (97), leading to the increased cell proliferation and cytokine release (97, 98). Ho et al. demonstrated that the tumoricidal effector functions of CD4^+^ T cells is inhibited in the presence of glucose deficiency, accompanying with the decreased IFN-γ and the increased TGF-β production. Further, phosphoenolpyruvate (PEP) was found to participate in sustaining T cell receptor-mediated Ca2^+^- NFAT (nuclear factor of activated T cells) signaling and effector functions. Up-regulation of PEP can activate the anti-tumor effect of T cells (99). In addition, compared with the naive T cells, the OXPHOS of in mitochondria is enhanced in activated T cells. Cytochrome c oxidase (COX), as a critical regulator of OXPHOS, also shows an improved activity (100). T cells activation induces a large increase in Gln import, and depletion of Gln blocks their proliferation and cytokine production (101). Gln can be partially replaced by nucleotides and polyamines (102), implicating Gln as an important source for biosynthetic precursors in active T cells. Moreover, L-Arginine is found to modulate CD3ζ expression and T cell function. T cells cultured in absence of L-arginine, present a sustained down-regulation of CD3ζ, exhibit a decreased cell proliferation and a significantly diminished production of IFN-γ, IL-5, and IL-10 (103–105).

FAO can function in regulating the balance between effector T cells (ETCs) and Tregs. It can promote Treg generation and inhibit their polarization toward ETCs (67). Gerriets showed that compared with Th17, Tregs have a higher expression of the FAO-related gene like carnitine palmitoyl transferase 1a (CPT1a) (106). FAO also participates in the activation and maintenance of CD8^+^ memory T cells. It was reported that IL-15, a cytokine critical for CD8^+^ memory T cells, regulates oxidative metabolism by promoting expression of CPT1a and control the bioenergetic stability of CD8^+^ memory T cells (107). Fatty acid synthesis is also critical to regulation of T cell function. Acyl-CoA carboxylase (ACC) is a rate-limiting enzyme in fatty acid synthesis. The absence of ACC was shown to inhibit the proliferation of CD8^+^ memory T cells (108). By inhibiting ACC, Berod et al. demonstrated that Th17 cells, but not Treg cells, depend on ACC1-mediated fatty acid synthesis for their development (109).

In *Plasmodium chabaudi* (*P. chabaudi*) infection model, down-regulating fatty acid synthesis by inhibiting ACC increases ETCs proliferation and strongly decreases peak parasitemia. It is noteworthy that this effect occurs only when fatty acid synthesis is inhibited during T cell priming (110). The most lethal complication of *Plasmodium falciparum* (*P. falciparum*) infection is cerebral malaria. Gordon et al. found that Gln analog 6-diazo-5-oxo-L-norleucine (DON) shows significant therapeutic effect. Within hours of DON treatment, mice showed blood-brain barrier integrity, reduced brain swelling, which is associated with the enhanced function of activated effector CD8^+^ T cells (111). This suggests that DON may regulate the immunity to alleviate the pathological changes in malaria infection. Compared with normal mice, Cysteine protease cathepsins B (Cat B)-deficient mice recover from *Leishmania major* (*L. major*) infection faster with decline of inflammation and decreased parasite burden. Further, it was showed that CD3^+^ T cells are the target cells for these effects, suggesting that Cat B can regulate T cell response during *L. major* infection (112). In addition, P-glycoprotein (P-gp) is a transporter protein involved in the transport of IL-2, IL-4, and IFN-γ in normal T cell (113). It was reported that *H. polygyrus* infection contributes to the inhibition of T cell function by elevating P-gp activity. The blockade of P-gp in the T cells leads to an impressive increase in T-cell proliferation and IL-4 cytokine release through the upregulation of NF-κB activation (114).

## B Cells

B cells are typically characterized by their ability to produce antibodies, function as secondary antigen-present cells, and produce various immunoregulatory cytokines. The regulatory B cells (Bregs) is now widely accepted as an important modulatory component of the immune system that suppresses inflammation. Notably, almost all B-cell subsets can be induced to form Bregs [As reviewed by Reference (115)]. In fact, the immunoregulatory function of Bregs can be mediated by production of cytokines such as IL-10 and TGF-β and ensuing suppression of T cells, by direct cell-cell interactions, and (or) by altering the immune microenvironment (116, 117). Several studies have revealed that Bregs largely expand in response to the infection of parasites like *L. major* and *Schistosoma* sp. and *E. granulosus* (17, 118, 119), indicating that the B cell subset plays an important role in anti-infectious immunity. In fact, TLR signaling is essential for triggering the expanding of Bregs (120). We previously reported that the ESPs of the larval *E. granulosus* induce the differentiation of Bregs *in vitro via* interacting TLR-2 (118). As mentioned above, *s*timulation with TLR agonists can activate DC through metabolic reprogramming (83, 84), we also found that PTEN, the key modulator of glucose and lipid metabolism (35), is significantly upregulated by the ESPs of the larval *E. granulosus*. Interestingly, the upregulation of PTEN expression mediated by ESPs seems to be dependent on TLR-2 signaling (118). Thus, it is possible that PTEN mediated metabolic reprogramming may participate in the Bregs differentiation induced by parasitic infection.

In summary, specific metabolic pathways can determine the differentiation and function of macrophage, DC, T and B cells, which provides a new perspective for elucidating the mechanism of immune regulation induced by parasites.

## Summary and Prospect

As one of important pathogens, several parasites have coevolved with human for a long history, and develop elegant and intricate immune escape strategies to manipulate the equilibrium of immune and metabolism in hosts. However, our understanding in regard to these underlying mechanism is still relatively superficial. Immunometabolism, the emerging discipline that integrates immunity and metabolism, provides a novel insight for exploring these scientific puzzles in Parasitology. In fact, immunity and metabolism interact with each other. For example, fibroblast growth factor 21 (FGF21) was reported to not only increase energy consumption and resist diabetes, but also maintain thymus structure and increase the number of thymic T cells (121). Leptin, a hormone secreted by adipocytes, can regulate the systemic energy metabolism, while it is also involved in regulating immunity and promoting the progression of autoimmune diseases, obesity-related cardiovascular diseases (122).

Emerging evidence has indicated that parasites or their derived molecules can reprogram the metabolic events in macrophage, DC, T and B cells of host, thereby modulating the anti-infective immunity and creating a promised or moderate pathophysiological state for the growth and development of parasites. However, only sporadic mechanistic investigations in the view of immunometabolism have been reported (27, 38). Several metabolites may be implicated in the underlying mechanism of parasitic infection and immunity. For example, SUCNR1, the succinate receptor, has recently been found to upregulate the expression of the markers associated with M2 macrophage in obesity (55). Immune responsive gene 1 (IRG-1, also named by Cisaconite decarboxylase 1, ACOD1), encodes the enzyme that catalyzes the cis-aconitate to produce itaconate in the TCA cycle (**Figure 1**). Recent studies have confirmed that ACOD1/itaconate axis links metabolism to immunity in macrophage (123), and has gained lots of interests in immunometabolism field and inflammatory disease models (124, 125). Interestingly, our microarray analysis in the fat tissues with larval *E. granulosus* infection also showed the upregulated levels of SUCNR1, succinodehydrogenase (SDH) and ACOD1 and enriched pathways such as Arginine metabolism and PPAR signaling pathway (67), which is consistent to the M2 polarization of macrophage post infection (**Figure 1**). These studies highlight the intimate interconnection between immune and metabolism of host and parasite. Further progress in identifying immunometabolic signaling and programming events is likely to provide a novel insight for clarifying the pathogenesis and host-parasite interplay.

Vaccination is an efficient means of combating infectious disease burden globally. However, routine vaccines for the world’s major human parasitic diseases do not yet exist. Lower protective effect is one of the main problems that cause the vaccine unavailability. Interestingly, several studies have showed that rigorous innate immune responses endue some hosts with the natural resistance against parasitic infection (126, 127). From the perspective of enhancing innate immunity, immunometabolism may open new opportunities to design a novel vaccine against parasitic diseases. For example, the mevalonate pathway is an indispensable step of intracellular cholesterol biosynthesis in antigen presenting cells (128). A recent study have uncovered that inhibition of this pathway can prolong antigen retention and heighten immune response of Th1 and cytotoxic T cells *in vivo* (129), indicating that the inhibitor can be a novel vaccine adjuvant. It is therefore believed to boost the protective rate of current parasitic vaccines. Furthermore, ACOD1/itaconate/SDH is demonstrated to be the key metabolic node for controlling the immune activation and tolerance in monocyte/macrophage (58, 125, 130). It is reasonable to speculate that targeting this pathway may enhance the innate immune response to resist the invading of parasites. In summary, immunometabolism, as an emerging integrated discipline, will benefit to develop several novel intervention strategies for preventing parasitic diseases in the future.

## Author Contributions

J-yC and J-kZ wrote the manuscript. WP revised the manuscript. All authors contributed to the article and approved the submitted version.

## Funding

Project support was provided in part by the National Natural Science Foundation of China (No. 81871670), Natural Science Foundation of Jiangsu Province (No. BK20201459) and Jiangsu QingLan Project. The funders had no role in study design, data collection and analysis, decision to publish, or preparation of the manuscript.

## Conflict of Interest

The authors declare that the research was conducted in the absence of any commercial or financial relationships that could be construed as a potential conflict of interest.
